# Chronic cannabis use and error awareness: The effect on learning from errors

**DOI:** 10.1371/journal.pone.0283158

**Published:** 2023-03-28

**Authors:** Gezelle Dali, Antoinette Poulton, Robert Hester

**Affiliations:** Melbourne School of Psychological Sciences, The University of Melbourne, Parkville, VIC, Australia; Federal University of Paraiba, BRAZIL

## Abstract

**Background:**

Cannabis is the third most commonly used drug worldwide, with studies suggesting a deleterious effect on some aspects of performance monitoring. It is unknown, however, whether diminished error awareness influences adaptive behaviour in cannabis users. Therefore, this study examined the effect of error awareness on learning from errors in cannabis users.

**Methods:**

Thirty-six chronic cannabis users (*M*_*age*_ = 23.81 years; female, 36%) and 34 controls (*M*_*age*_ = 21.53 years; female, 76%) completed a Go/No-Go task that allowed participants to learn from errors and adapt their behaviour. Multilevel models were specified to determine whether the effect of error awareness on learning from errors differs between cannabis users and controls, and whether cannabis-use measures predict error correction while accounting for error awareness.

**Results:**

While error awareness and correction rates did not differ between the groups, there was a significant effect of age of use onset on error correction in cannabis users. Further, the effect of error awareness was dependent on age of onset, and cannabis use-related frequency and harm. That is, cannabis users reporting an earlier age of regular use or scoring higher on the cannabis use index were less likely to perform correctly following an aware error.

**Conclusion:**

It appears overall cannabis use might not be tightly coupled to behavioural indices of performance monitoring. There is evidence, however, that aspects of cannabis use predict impairments in learning from errors that may be associated with treatment outcomes.

## 1. Introduction

Error processing is an integral component of behavioural regulation. This system facilitates behaviour by employing cognitive control mechanisms to implement behavioural modifications [1–3]. Notably, impaired error monitoring and subsequent behavioural regulation are purported to underlie several clinical conditions [4–6]. Substance misuse–that is, regular or chronic subclinical use of a substance–has been found to be associated with difficulties monitoring behaviour, contributing to failed efforts to reduce use and an increased risk of transition to dependence [7–9]. Additionally, compromised error processing in substance use disorder populations has been reported to interfere with users’ treatment retention and their ability to understand and engage in intervention programs [10]. Thus, there is a clear imperative to understand the error processing mechanisms contributing to poor behavioural regulation in individuals at risk of substance use disorder.

Cannabis is the third most commonly used drug worldwide, providing impetus for investigating the potential ramifications of long-term use on error monitoring processes [11]. Despite the clear imperative, however, there is a severe paucity of research on error processing in chronic cannabis users. In the few studies within this area of research, chronic cannabis users have largely not been found to exhibit differences in overall accuracy or reaction time [12–16], including post-error performance [17]. There is some evidence, however, to suggest that indices such as age of onset may be predictive of impaired performance. For example, Battisti, Roodenrys [14] found age of onset to predict reduced accuracy on incongruent trials in a Stroop task, indicating that regular exposure to cannabis at an earlier age may impinge on cognitive processes at a behavioural level. While the primary interest here is on behavioural findings, given the dearth of research in this area, it is worth considering neurobiological accounts which can be used to surmise the effect of long-term cannabis use on error monitoring processes. Specifically, models on error processing have posited that midbrain dopaminergic neurons are crucial to error-based learning by responding with a temporary decrease in firing rate to outcomes that are worse than expected [18]. This in turn disinhibits the anterior cingulate cortex (ACC), resulting in faciliatory adaptive behaviour. Importantly, cannabis users have been shown to display long-term dopaminergic hypoactivity [19–21] in conjunction with reduced error-related ACC activity [16, 22–24], even in the absence of behavioural deficits. Since error monitoring is dependent upon the mesencephalic dopaminergic system, it is likely that chronic cannabis use has a deleterious effect on this process.

Of particular interest, cannabis users have been found to demonstrate reduced awareness of errors in the presence of intact inhibitory control [23]. This is particularly pertinent as we have recently found that error awareness facilitates adaptive behaviour; namely the ability to learn from errors [25]. Impaired error awareness may explain, in part, why individuals who exhibit substance misuse and/or dependence are less sensitive to the outcomes of their actions and are therefore more likely to repeat maladaptive behaviours, including the maintenance of drug use [26]. That is, failure to learn from errors may contribute to a cycle of dysfunctional behaviours. Indeed, previous research has shown that chronic cannabis users display compromised task adaptation in response to negative feedback [24]. Further, individuals who use cannabis have been found to demonstrate a reduced ability to learn from errors, as indicated by poorer recall and a lower error-correction rate relative to controls [22]. Notably, however, studies have not explored the relationship between diminished error awareness and subsequent adaptive behaviour in chronic cannabis users. The deficit in error awareness may be an important contributor to the learning impairments observed in cannabis users, however, this remains to be systematically tested.

The current study sought to examine error awareness in chronic cannabis users using a paradigm that allowed participants to learn from errors and subsequently adapt their behaviour. A motor Go/No-Go response inhibition task was employed whereby participants commit aware and unaware errors. The task allowed for error awareness to be linked to adaptive behaviour (i.e. learning from errors). To afford participants the opportunity to intently display corrective action, task presentation was highly contingent on errors. That is, inhibition performance determined the sequence of No-Go trials such that there was a high probability that an erroneous No-Go trial was succeeded by a No-Go trial that presented the same No-Go stimulus that was failed. Thus, the task allowed participants to correct their commission error on a subsequent post-error No-Go trial. It was hypothesised that cannabis users would demonstrate poorer error awareness. Further, we predicted that correction of errors would depend on error awareness, thus resulting in poorer error correction in chronic cannabis users.

## 2. Material and methods

### 2.1. Participants

Thirty-six cannabis users (*M* = 23.81 years, *SD* = 5.36, age range, 19–44 years) and 34 control participants (*M* = 21.53 years, *SD* = 2.95, age range, 18–30 years) were recruited from The University of Melbourne campus and experimenter networks. Participants were excluded if they self-reported having a substance use disorder as the primary interest here was the effect of sub-clinical use. All participants provided informed consent and were reimbursed for participation. The study received approval by The University of Melbourne’s Human Research Ethics Committee for meeting the standards prescribed by the Australian National Health and Medical Research Council. Participants were classified as cannabis users if they reported using cannabis regularly (weekly basis) during the past year and as a non-user if they reported using fewer than four times during the past year and less than 10 times in their lifetime [14]. It should be noted that more than half of participants (52.8%) in the cannabis group reported using cannabis near-daily or daily for the preceding 12 months. The majority of non-users (73.5%) reported having never used cannabis, with most of the remaining participants reporting using over one year ago. Two control participants reported use in the previous year, with one reporting 1–5 lifetime uses and one reporting 6–10 lifetime uses.

### 2.2. Measures

#### 2.2.1. Cannabis use disorder identification test–Revised (CUDIT-R)

The CUDIT-R [27] is a 10-item measure that assesses cannabis intake and misuse over the preceding 6 months. Scores of 8 or more indicate hazardous use, while scores of 12 or more suggest potential cannabis use disorder. The CUDIT-R has been found to demonstrate excellent internal consistency (Cronbach’s alpha = .91) [27].

#### 2.2.2. Daily sessions, frequency, age of onset, and quantity of cannabis use inventory (DFAQ-CU)

The DFAQ-CU [28] is an inventory designed to assess frequency, age of onset and quantity of cannabis use. Item scores are transformed to *z-*scores prior to calculating the mean of the six factors: daily sessions, frequency, age of onset, marijuana quantity, concentrate quantity, edibles quantity. Table 2 presents these factors in unstandardised form, however, where a factor was used in analysis, the standardised form of the variable was used. The factors have demonstrated good internal consistency ranging from .69 (daily sessions) to .95 (frequency) [28].

#### 2.2.3. Timeline Follow-back (TLFB)

The TLFB [29] is a retrospective measure of drug use. Participants indicated the amount of cannabis consumed (in grams) on each of the 30 days. Participants were able to refer to an image of various quantities of marijuana relative to a $20 Australian note to assist in their estimations.

#### 2.2.4. Alcohol, Smoking and Substance Involvement Test (ASSIST)

The ASSIST [30] evaluates patterns of nicotine, alcohol, cannabis, cocaine, inhalant and other substance use in the preceding 3 months. For alcohol, scores of 11–26 indicate moderate risk of abuse and dependence, while scores of 4–26 indicate moderate risk for all other substances. The ASSIST has demonstrated good internal consistency across all substance domains (Cronbach’s alpha = .77-.94).

#### 2.2.5. Patient Health Questionnaire– 9 (PHQ-9)

The PHQ-9 [31] is a 9-item screening measure that evaluates symptoms of major depressive disorder over the preceding 2 weeks. Scores more than or equal to 10 indicate moderate depression. The PHQ-9 has shown good internal consistency (Cronbach’s alpha = .89) [31].

#### 2.2.6. Generalised Anxiety Disorder– 7 (GAD-7)

The GAD-7 [32] is a 7-item screening tool that assesses symptoms of generalised anxiety disorder over the preceding 2 weeks. Scores more than or equal to 10 indicate moderate anxiety levels. The GAD-7 has demonstrated excellent internal consistency (Cronbach’s alpha = .92) [32].

### 2.3. Behavioural task

**Fig 1:**
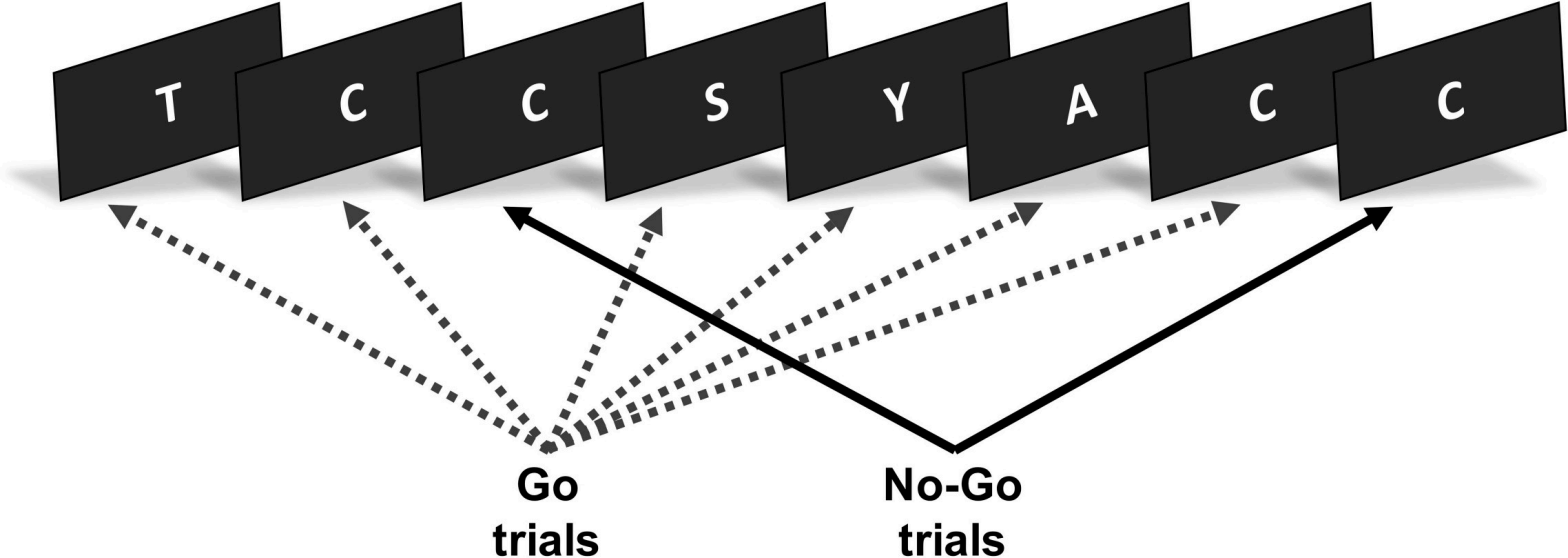
Learning from inhibition errors task. The task presented a serial stream of letters. The figure presents an example of a No-Go trial (the repetition of the letter ‘C’) followed by the same stimulus on the following No-Go trial (consecutive repetition of the letter ‘C’). This sequence will only occur when participants have not inhibited their response on the first No-Go trial.

**Fig 2:**
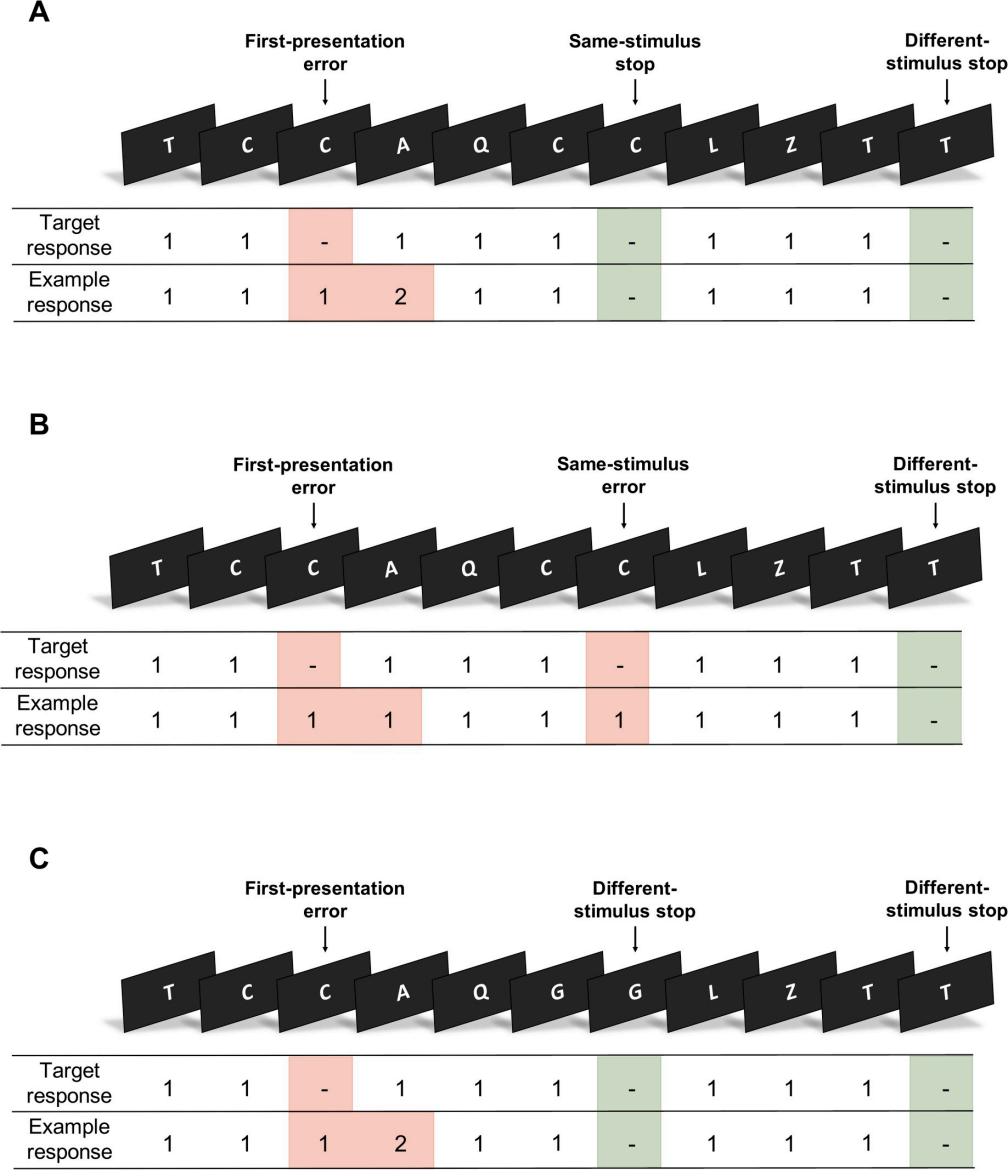
Classification of No-Go events. Participants responded to each letter using a left button press (‘1’) and withheld their response whenever a letter was presented consecutively (No-Go trial). To indicate error awareness, participants were required to forgo making a standard ‘Go’ response and to instead execute a right button press (‘2’) on the trial following the commission error. No-Go trials that presented the same stimulus as the previous No-Go trial were classified as ‘same-stimulus’ No-Go trials. Those that presented a different stimulus were classified as ‘different-stimulus’ No-Go trials. No-Go trials were categorised based on performance (errors shaded red, correct stops shaded green). For 75% of first-presentation No-Go errors, the next No-Go trial was a same-stimulus No-Go trial (A). For the remaining 25% of first-presentation No-Go errors, the next No-Go trial was a different-stimulus No-Go trial (C). The No-Go trial following a correct stop was always a different-stimulus No-Go trial (A, C).

The Learning from Inhibition Errors task is a motor Go/No-Go response inhibition paradigm [25]. The task was programmed and delivered using E-Prime software (version 1.1, Psychology Software Tools). Each task trial presented a random letter from the English alphabet (Fig 1). Participants were required to make a left button press for each letter in the sequence (Go trials), unless the letter was presented consecutively (No-Go trials). On such trials, participants were required to withhold their response. To indicate error awareness, participants were trained to forego making a standard ‘Go’ response on the Go trial following an incorrect No-Go response (commission error) and to instead execute a right button press (Fig 2).

Participants were instructed that the trial sequence was influenced by their performance on No-Go trials. A correct No-Go response guaranteed that the next No-Go trial presented a different (random) letter stimulus. For 75% of incorrect No-Go responses, the next No-Go trial presented the same letter stimulus (i.e. a context-specific condition). The task ensured that the letter would not appear as a Go trial between the first-presentation error and the following No-Go trial. This task design incentivised participants to encode the letter during an error as it enabled them to predict the highly probable appearance of the next No-Go trial and therefore avoid making a consecutive erroneous response. If a participant failed to withhold their response on a second consecutive No-Go trial, the No-Go trial following the second error presented a different letter of the alphabet. This ensured that a maximum of two consecutive No-Go trials would present the same letter. Participants were instructed to respond as quickly and as accurately as possible.

Participants were initially administered a practice version of the task comprising 120 trials (110 Go trials, 10 No-Go trials). The full task comprised five blocks of 265 trials (225 Go trials, 40 No-Go trials). All stimuli were presented for 700ms followed by a 600ms inter-stimulus interval. No-Go trials were distributed pseudo-randomly throughout the serial presentation of Go trials. The number of Go trials separating No-Go trials ranged between four and 12 (*M =* 6.60; *SD* = 1.61). Testing conditions were kept constant across participants.

No-Go trials were classified according to whether they presented the same letter stimulus as the previous No-Go trial (Fig 2). No-Go trials that presented the same stimulus as the previous No-Go trial were classified as ‘same-stimulus’ No-Go trials (i.e. a context-specific condition). Those that presented a different stimulus were classified as ‘different-stimulus’ No-Go trials. Based on participant performance, No-Go trial events were classified into correct responses, unaware and aware errors. Correct No-Go responses (stops) were those on which the participant successfully withheld a response, while incorrect No-Go responses (commission errors) were those on which the participant did not inhibit a response. The second in a pair of consecutive No-Go errors (of which the second No-Go trial was a same-stimulus trial) was classified as a same-stimulus No-Go error. If the participant responded with a left button press on the No-Go trial and again on the following Go trial, the commission error was classed as unaware. Any deviation from this was classified as an aware error.

### 2.4. Data analysis

Data analysis was undertaken using the programming language R [33]. Assumptions were tested, and non-parametric analyses were computed under violations of normality. *P*-values were adjusted using Holm procedures. Alpha was set to .05 for all analyses. It should be noted that three participants reported a past head injury. Exclusion of these participants did not alter our results and thus we opted to report our analyses with these participants included. All data and R code required for the current results have been made publicly available online at the Open Science Framework (https://osf.io/4dxhm/).

#### 2.4.1. Effect of cannabis use and error awareness on future performance

To assess the difference in post-error performance between cannabis users and controls, while accounting for error awareness, a mixed effects logistic model was computed using the ‘glmer’ function from the *lme4* package [34]. The resulting data had a multilevel structure such that erroneous trials were nested within blocks which were nested within individuals. The interaction between group (cannabis users and controls) and awareness of a given erroneous No-Go trial (aware and unaware) served as a fixed effect used to predict inhibition performance on the subsequent No-Go trial (correct No-Go response and incorrect No-Go response). We also included the interaction between awareness and trial type (first-presentation errors followed by same-stimulus No-Go trial and different-stimulus No-Go trial) as a fixed effect, in order to verify under what conditions error awareness subserves adaptive behaviour.

A second model was computed for the cannabis group in order to determine the effect of cannabis-use measures on error correction, when accounting for error awareness. This model included only first-presentation errors that were followed by a same-stimulus No-Go trial, as the interest here was on corrective performance under context-specific conditions (i.e. when a situation that was previously failed is re-encountered). In order to avoid multicollinearity, a standardised cannabis use index (CUI) was created using a method employed by previous studies [35, 36]. To obtain the CUI, the mean of *z*-scores for the following four correlated cannabis-use measures was computed: TLFB-reported frequency of use (days of use over 30-day period), DFAQ-CU frequency score, ASSIST cannabis score and CUDIT score. The interaction between awareness and the CUI was used as a fixed effect to predict inhibition performance on the subsequent No-Go trial (correct No-Go response and incorrect No-Go response). The interaction between DFAQ-CU age of use onset and awareness was included as a second fixed effect as previous studies have found preliminary evidence of an association between inhibition performance and age of onset [14, 23]. In line with Cuttler and Spradlin [28], age of onset was calculated by taking the mean of *z*-scores for four DFAQ-CU items which assess age of first use, age of regular cannabis use, age of daily or near-daily cannabis use, and frequency of cannabis use before the age of 16.

A binominal distribution with a logit function was specified in both models, and subject and block were included as nested random effects [37, pp. 553–586]. Error number was not included as a nested random effect due to near zero variance. *P-*values were estimated using the *lmerTest* package [38]. Odds ratios were calculated for the fixed effects and bootstrap confidence intervals were derived using parametric bootstrapping.

#### 2.4.2. Reaction time adjustments

Switching to the awareness button typically results in abnormally fast reaction times on the Go trials proceeding the error [25]. Response speed adjustments following erroneous trials were therefore determined by calculating the difference in reaction time for each Go trial proceeding the No-Go by at least three trials (up to the next pre-No-Go trial) and the Go trial immediately preceding the No-Go trial (a subtraction of the pre-error Go reaction time from the post-error Go reaction time). Erroneous No-Go trials (and the proceeding post-error trials) that followed pre-No-Go trials on which the participant did not make a response were excluded from analysis.

A mixed effects model was estimated using the ‘mixed’ function from the *afex* package [39]. The model was specified to compare post-No-Go reaction time adjustments across groups and response types and included two fixed effects and their interactions: group (cannabis users and controls) and response (correct, aware and unaware errors). Subject and error number were included as nested random effects. Block was not included as a nested random effect due to near zero variance. Random slopes were not included in the model as they did not improve model fit. Post-hoc tests were undertaken using the *emmeans* package [40]. Marginal means and standard errors were computed using the ‘emmeans’ function and pairwise comparisons were conducted using the ‘pairs’ function.

## 3. Results

### 3.1. Demographics and drug use

Demographic and drug use data are reported in Table 1, while cannabis use indices can be found in Table 2. Cannabis users and controls differed with respect to sex, with significantly more cannabis users likely to be males, χ^2^ = 9.97, *p* = .014. With regard to other drug use, cannabis users were found to score higher on tobacco, W = 1021.5, *p* < .001, amphetamine, W = 837.5, *p* = .013, and hallucinogenic use, W = 946, *p* < .001. No differences were found on measures of age, educational attainment, other drug use or mental health.

**Table 1:** Demographic and survey measures of drug use and mental health.

	Cannabis (*n* = 36)	Controls (*n =* 34)	*p-*value
Age (years)	23.81 (5.86)	21.53 (2.95)	.251
Sex (F:M)	13:23	26:8	.016
Highest level of education			.094
Primary school	2.8% (*n* = 1)	0% (*n* = 0)	
Secondary school	8.3% (*n* = 17)	73.5% (*n* = 25)	
Trade qualification	0% (*n* = 0)	8.8% (*n =* 3)	
Complete bachelor’s degree	22.2% (*n* = 8)	8.8% (*n* = 3)	
Postgraduate degree	27.8% (*n* = 10)	8.8% (*n* = 3)	
CUDIT	11.28 (6.05)	0.41 (1.91)	< .001
ASSIST			
Tobacco	14.06 (10.53)	3.03 (7.33)	< .001
Alcohol	11.56 (8.04)	8.09 (7.10)	.275
Cannabis	16.25 (7.96)	0.85 (2.19)	< .001
Cocaine	0.86 (1.78)	1.15 (4.36)	> .999
Amphetamines	2.67 (5.00)	0.85 (2.89)	.014
Inhalants	1.06 (2.11)	0.21 (0.91)	.131
Sedatives	0.92 (2.05)	0.41 (1.74)	.454
Hallucinogens	3.36 (4.20)	0.15 (0.61)	< .001
Opioids	0.56 (1.99)	0 (0)	.454
PHQ-10	4.94 (4.28)	5.55 (3.88)	> .999
GAD-7	3.92 (4.29)	4.55 (4.62)	> .999

*Note*. Values are expressed as means (standard deviation) unless otherwise noted. The *p*-value indicates whether the groups differed significantly. CUDIT, Cannabis Use Disorder Identification Test; ASSIST, Alcohol, Smoking and Substance Involvement Screening Test; PHQ-10, Patient Health Questionnaire– 10, GAD-7, Generalised Anxiety Disorder– 7.

**Table 2:** Cannabis use indices.

	Cannabis (*n* = 36)	Controls (*n* = 34)^a^
Age of first use	16.97 (2.06)	17.67 (3.12)^b^
Cannabis use (years)	6.83 (5.12)	-
Regular cannabis use (years)	4.88 (5.43)	-
Recency of use (days)	1.69 (1.92)	
Cannabis use frequency over last year		
No use	0%	94.1% (*n* = 32)
Less than four times a year	0%	5.9% (*n* = 2)
Two or three times a month	8.3% (*n* = 3)	0%
Once a week	8.3% (*n* = 3)	0%
Twice a week	30.6% (*n* = 11)	0%
Three or more times a week	25.0% (*n* = 9)	0%
Daily	27.8% (*n* = 10)	0%
DFAQ-CU–Age of onset	16.12 (1.99)	-
DFAQ-CU–Daily sessions	1.61 (0.88)	-
DFAQ-CU–Frequency	5.84 (2.10)	-
DFAQ-CU–Marijuana quantity	1.65 (1.57)	-
DFAQ-CU–Concentrate quantity	0.35 (1.71)	-
DFAQ-CU–Edible quantity	3.08 (3.92)	-
TLFB–Days of use over last 30 days	16.76 (9.69)	0.03 (0.16)
TLFB–Use over last 30 days (grams)	19.10 (25.35)	0.04 (0.25)

*Note*. Values are expressed as means (standard deviation) unless otherwise noted. DFAQ-CU, Daily Sessions, Frequency, Age of Onset, and Quantity of Cannabis Use Inventory; TLFB, Timeline Follow-back.

^a^DFAQ-CU indices are not reported for controls as these measures can only be computed for users. The instrument does not require participants to respond to questions if they report having never used cannabis.

^b^This value pertains to participants who reported ever having used cannabis (*n* = 9).

### 3.2. Performance measures

Since the groups differed on sex, and tobacco, amphetamine and hallucinogenic use, a series of Wilcoxon rank-sum tests and Spearman correlations were conducted to determine whether individual differences in these variables were related to differences in our dependent variables. These variables were not found to differ or correlate significantly (*p*s *>* .05) with any task measure and were therefore not included as covariates in subsequent analyses.

Performance indices are summarised in Table 3. Error awareness, W = 723, *p* = .194, and inhibition performance, W = 539.5, *p* = .397, were not found to differ between cannabis users and controls. There was no difference in reaction time between correct Go trials, aware errors and unaware errors, *F*(1.43, 84.17) = 2.94, *p* = .075, η_p_^2^ = .02. Further, there was no between-group difference in reaction time, *F*(1, 59) = 2.77, *p* = .101, η_p_^2^ = .03, nor interaction effect between group and trial type (correct Go, aware and unaware error) on reaction time, *F*(1.43, 84.17) = 0.37, *p* = .690, η_p_^2^ = .003. There was also no effect of recency of use on error awareness, *F*(6, 29) = 1.51, *p* = .211, η_p_^2^ = .23, or inhibition performance, *F*(6, 29) = 2.00, *p* = .097, η_p_^2^ = .29.

**Table 3:** Learning from inhibition errors task performance indices.

	Cannabis (*n* = 36)	Controls (*n* = 34)
Error awareness (% aware)	80.85 (31.11)	83.60 (23.06)
Go RT (ms)	327.94 (51.47)	337.01 (59.82)
Overall inhibition accuracy (% correct)	64.2 (19.01)	67.31 (19.91)
Post-error inhibition accuracy (% correct)		
Same-stimulus No-Go trial	75.16 (19.11)	78.29 (20.79)
Different-stimulus No-Go trial	50.84 (29.00)	54.72 (24.67)
Post-correct inhibition accuracy (% correct)	62.56 (17.96)	63.61 (22.22)
Error RT (ms)		
Aware error	358.62 (110.82)	367.35 (121.57)
Unaware error	352.01 (104.31)	393.64 (117.26)

*Note*. Values are expressed as means (standard deviation). RT, reaction time.

### 3.3. Learning from errors

Results of the mixed-effects logistic regressions are summarised in Table 4. Model 1 investigated the difference in post-error performance between cannabis users and controls. Model 1 showed a significant predictive effect of trial type on performance, β = 0.81, 95% CI, [0.38, 1.25], OR = 2.26, 95% CI, [1.47, 3.48], such that correct inhibitions following an error were more likely to occur on same-stimulus No-Go trials. As expected, this effect was found to depend on awareness, β = 0.56, 95% CI, [0.06, 1.06], OR = 1.76, 95% CI, [1.07, 2.89]. That is, correct inhibitions on same-stimulus No-Go trials more frequently followed an aware error than unaware error. There was, however, no main effect of awareness or group, and no significant interaction between awareness and group on future performance. Model 2 investigated the effect of cannabis-use measures on error correction in cannabis users. Consistent with Model 1, our findings for Model 2 indicate that under context-specific conditions whereby a No-Go error was followed by a same-stimulus No-Go trial, there was a significant effect of awareness on future performance, β = 0.71, 95% CI, [0.29, 1.14], OR = 2.04, 95% CI, [1.33, 3.12]. There was also a significant effect of age of onset on performance, β = 0.82, 95% CI, [0.07, 1.58], OR = 2.28, 95% CI, [1.07, 4.86]. That is, those reporting an earlier onset of regular cannabis use were less likely to perform correctly on a No-Go trial following a No-Go error. Notably, a significant interaction was found between awareness and the cannabis use index (CUI), β = -0.67, 95% CI, [-1.21, -0.14], OR = 0.51, 95% CI, [0.30, 0.87], and between awareness and age of onset, β = -0.62, 95% CI, [-1.22, -0.02], OR = 0.54, 95% CI, [0.29, 0.97]. These results indicate that cannabis users scoring higher on the CUI or reporting an earlier age of onset were less likely to perform correctly following an aware error. No main effect of CUI score was found.

**Table 4:** Mixed effects logistic models predicting post-error inhibition performance.

						OR 95% CI	
	Fixed effects	β	*SE*	*z*	OR	Lower	Upper
Model 1							
	Intercept	0.29	0.32	0.90	-	-	-
	Awareness	-0.06	0.29	-0.20	0.94	0.54	1.66
	Group	-0.17	0.35	-0.50	0.84	0.43	1.66
	Trial type	0.81**	0.22	3.67	2.26	1.47	3.48
	Awareness:Group	-0.04	0.27	-0.16	0.96	0.57	1.62
	Awareness:Trial type	0.56*	0.25	2.22	1.76	1.07	2.89
Model 2							
	Intercept	0.72	0.25	2.93	-	-	-
	Awareness	0.71**	0.22	3.29	2.04	1.33	3.12
	CUI	0.64	0.34	1.89	1.15	0.97	3.64
	Age of onset	0.82*	0.39	2.14	0.86	1.07	4.86
	Awareness:CUI	-0.67*	0.27	-2.48	0.56	0.30	0.87
	Awareness:Age of Onset	-0.62*	0.31	-2.03	1.76	0.29	0.98

*Note*. CUI, cannabis use index; CI, confidence interval. Model 1, *N* = 70; Model 2, *N* = 36. Both models include subject and block as nested random effects.

**p < .010;

*p < .050.

### 3.4. Reaction time adjustments

With regard to reaction time adjustments following No-Go trials, we found a main effect of response type (correct, aware and unaware errors) on post-No-Go speed, *F*(2, 15478.18) = 21.93, *p* < .001. That is, reaction time was found to be slower following aware errors, *t*(39) = 3.68, *p* = .002, and unaware errors, *t*(179) = 5.83, *p* < .001, relative to correct responses. Reaction time was also greater following unaware errors compared to aware errors, *t*(236) = 3.80, *p* < .001; a result that is typical in error awareness tasks. In addition, we found a main effect of group on post-No-Go adjustments, *F*(1, 140.74) = 4.92, *p* = .030, such that cannabis users showed greater slowing following No-Go trials than controls, *t*(98.4) = 2.22, *p* = .027. No significant interaction effect was found, *F*(2, 15478.18) = 2.89, *p* = .060.

## 4. Discussion

This study sought to examine the effect of chronic cannabis use on error awareness and subsequent adaptive behaviour. Contrary to expectations, error awareness was not found to differ between cannabis users and controls. While error correction was dependent on error awareness under context-specific circumstances, there was no difference between the groups with regard to error correction. We did, however, find evidence in support of an effect of age of onset on error correction in cannabis users. That is, cannabis users reporting an earlier age of regular use were less likely to correct an error. Notably, the effect of error awareness was dependent on age of onset and the cannabis use index, such that cannabis users reporting an earlier age of onset or scoring higher on the cannabis use index were less likely to perform correctly following an aware error.

Although it has previously been shown that chronic cannabis users exhibit reduced error awareness compared to controls [23], the present study did not find evidence in support of this effect. Moreover, there was no evidence for a difference between the groups on accuracy and reaction time. These findings are largely consistent with previous reports of no differences in other indices of behavioural performance despite altered neural functioning [12–16]. These results suggest a possible dissociation between neural changes and behavioural impairments. This may be attributed to the presence of compensatory networks, where equivalent cognitive performance requires additional, alternative neural activity [14, 41]. It is also plausible that behavioural tasks are not sufficiently sensitive to detect subtle performance changes in non-dependent populations [36]. While we cannot discount the possibility that dependent users may show impairments, it is worth noting that similar findings have been reported in individuals with cannabis use disorder [17]. Regardless, given the sparsity of findings on error awareness, there is a clear need for further replication of the current results, particularly with longer-term cannabis users.

The absence of evidence for a difference in error correction between cannabis users and controls may be attributed to the increased post-No-Go slowing observed in cannabis users. That is, cannabis users may have adopted a conservative response strategy following No-Go trials in order to facilitate their post-No-Go performance. While this pattern of post-No-Go slowing in cannabis users has not previously been recorded, there is some evidence of greater post-error activity in the form of larger error positivity (Pe) amplitudes [12]. Therefore, it is plausible that cannabis users may need to devote greater cognitive resources in order to avoid error commission. The additional attention paid by cannabis users to the task may have manifested as increased post-No-Go slowing. It is also worth considering, in light of non-functional accounts of post-error slowing [42, 43], that slowing following No-Go trials may alternatively reflect greater reactivity in chronic cannabis users. Further exploration is ultimately necessary to clarify the robustness of this finding.

Interestingly, we found evidence to suggest that–in cannabis users–error correction was predicted by age of onset of cannabis use, with an earlier age of onset associated with poorer error correction. Further, the effect of error awareness on correction was found to depend both on the age of onset and overall cannabis-related frequency and harm. While previous studies have discerned poorer learning from errors in chronic cannabis users [22, 24], our results suggest that this relationship may be dependent on factors beyond cannabis use status alone. Age of onset, in particular, has previously been found to be associated with poorer behavioural performance in cannabis users [14, 44]. A clinical implication of these findings is that earlier use of cannabis may be an important predictor of impairments in learning from errors that may be associated with treatment outcomes.

One consideration is whether the present sample exhibits similar cannabis use characteristics to previous cohorts. The Australian Institute of Health and Welfare indicates that, among 18-29-year-olds who have used cannabis in the preceding year, approximately 30% have used once a week or more, and a further 15% use once a month [45]. Our cannabis-using group demonstrated greater frequency of use with the majority of individuals using daily, or near-daily for the preceding 12 months. Relative to Hester, Nestor [23] and Carey, Nestor [22], the current sample were found to have a similar age of onset (16.97 years in the current study compared to 16.4 years and 15.97 years, respectively), and comparable days of use in the last month (16.76 days compared to 19.2 days and 20.8 days, respectively). It is worth noting that our inclusion criteria required participants to have consumed cannabis regularly for at least the preceding year. Regardless, on average, our sample demonstrated a length of regular use exceeding regular use criteria adopted by previous studies [e.g. 12, 22, 23]. It appears, therefore, that the characteristic use of cannabis in this sample does not necessarily underlie the absence of a difference in error awareness and error correction between users and non-users.

This study is not without limitations. Given that previous work has demonstrated impaired neural correlates of error processing in chronic cannabis users in the absence of behavioural deficits [12, 14], we surmised that there may be a dissociation between neural changes and behavioural impairments. In order to accurately infer the presence of compensatory networks, however, our behavioural findings would ideally be supplemented with functional neuroimaging findings. Indeed, abnormalities in subclinical samples are typically not as pronounced as in clinical populations, thus neuroimaging techniques may reveal deficits not detected at a behavioural level [46]. An additional limitation is our reliance on self-report measures. While research supports the validity of self-report measures of cannabis use [47], without a structured clinical assessment we cannot definitively rule out the possibility that some participants may have satisfied DSM-5 criteria for cannabis use disorder. Finally, while our use of linear mixed models affords more statistical power than traditionally adopted analyses of variance [48], it is possible that the total number of No-Go trials in each category (i.e. aware and unaware errors by trial type) may have undermined the power of the current study. In particular, there were substantially fewer first-presentation errors followed by a different-stimulus No-Go trial than a same-stimulus No-Go trial. Further exploration of the current results is thus required.

In sum, the present study addresses the scarcity of research on error awareness in cannabis users by exploring the relationship between error awareness and learning from errors. The current results suggest that overall cannabis use might not be tightly coupled to behavioural indices of performance monitoring such as error awareness, however, there is evidence that aspects of cannabis use may predict impairments in learning from errors that are plausibly associated with intervention outcomes.
